# Comparison of tigers' fecal glucocorticoids level in two extreme habitats

**DOI:** 10.1371/journal.pone.0214447

**Published:** 2019-04-10

**Authors:** Sergey V. Naidenko, Mikhael A. Berezhnoi, Vinod Kumar, Govindhaswamy Umapathy

**Affiliations:** 1 A.N.Severtsov Institute of Ecology and Evolution, Moscow, Russia; 2 Moscow State Agricultural University, Moscow, Russia; 3 CSIR-Laboratory for the Conservation of Endangered Species, Centre for Cellular and Molecular Biology, Hyderabad, India; Banaras Hindu University, INDIA

## Abstract

Application of different antibodies and extraction methods results in a wide range of steroid metabolite concentrations obtained during noninvasive hormones monitoring. It makes regional comparisons of steroid concentration very difficult. We compared three methods for extraction of glucocorticoids metabolites in tiger feces to examine correct stress level in Bengal and Amur tigers in India and Russia respectively. The results obtained with three different extraction methods correlate with each other positively and significantly. The highest concentration of fecal glucocorticoids metabolites (FGCM) was found after the extraction of wet feces samples with 90% methanol. The level of FGCM was significantly higher in Bengal tigers in India than in Amur tigers in Russian Far East. The reasons might be related to tigers’ density or anthropogenic pressure.

## Introduction

Application of non-invasive methods of monitoring the hormonal and physiological status of wildlife has become a common and widespread approach over the last years [1–3], most frequently used for mammalian species [4–6]. Feces [7], urine [8] and hairs [9] are the most common substrates for this kind of analysis. The analysis helps to monitor the level of androgens in animals [10–11], to identify estrous or ovulation time in females [12], to diagnose pregnancy [3], to estimate food availability [13] and stress/welfare in individuals [14]. This non-invasive hormone assay method has been successfully used to examine reproductive status of wild animals for many breeding programs [15–17]. Later noninvasive methods of hormone monitoring were used to estimate the stress level of animals and the factors affecting it [9, 13].

Wide range of methods has been developed to estimate stress/ welfare of different cat species as [7, 12, 14–16]. Steroid hormones are excreted from the felids’ body mainly in feces (80–90%) [18] which ensures the choice of substrate for noninvasive hormones monitoring by different researchers. Several studies on felids hormones level in the wild were conducted over the last decade [19–23].

However, comparative estimation of the level of the same hormones at different study sites or in different countries is still very rare, since it is very difficult to conduct such experiment. For example, application of different antibodies (to cortisol [21], corticosterone [24], 11-oxoethiocholanolone [18] in different labs to measure stress in felids non-invasively makes the comparison impossible. Even the application of antibodies to the same antigen may show different concentrations in blood and especially in feces (two-three folds) [10]. These differences may increase due the variety of extraction methods in different labs and in field studies [7, 24, 25]. With different antibodies in the EIA and different extraction method will not allow normal comparison of hormone data for conservation management of wild animal. Thus using a common antibody and method of extraction would help in comparing hormone data in a species among the ranging countries.

Tiger (*Panthera tigris*) is one of the largest cats in the world. Its number has decreased dramatically over the 20^th^ century (down to 3500 individuals in the wild) [26]. At present, the range of this species is very fragmented, while previously it covered almost all of southern Asia from the Caucasus to Bali (Indonesia) [26–29]. More than half of the present tiger population inhabits India, the Amur tiger (P. tigris altaica) being the most isolated northern subspecies. Tiger as a species has evolved in south-eastern Asia [30] and adapted well to habitats like the jungle or open areas with tall grass. At the Russian Far East Amur tigers reside in extreme conditions: winter temperatures reaching -40°C and high snow levels (sometimes up to 100 cm). Amur tigers have much bigger home ranges (20 times) in comparison to home ranges of Bengal ones (*P*. *tigris tigris*) [31, 32] which is determined mainly by prey density in these regions [33]. At the same time anthropogenic effect is much higher in India where the density of human population is much higher than in Russian Far East.

Noninvasive studies of tigers’ hormonal status in the wild were conducted both in India and in Russia [21, 23]. In India these studies showed significant differences in levels of fecal glucocorticoids metabolites (FGCM) at different study sites (tiger reserves) and individual differences in FGCM level depending on habitats. A correlation between the FGCM level and reproductive ability of female tigers were also discovered [23]. In Russia a negative correlation was discovered between the FGCM level of tigers and winter air temperatures [34]. The most pronounced increase of FGCM was observed with the air temperature below -10°C.

This study aimed to compare FGCM level in Amur and Bengal tiger in winter period, and to compare FGCM in the same samples extracted by different methods to estimate the effect of extraction procedure on the concentration of FGCM.

## Methods

Amur tiger samples were collected in January-February of 2015 at the Russian Far East over the whole range during the total tiger snow survey. All foresters (scientists, volunteers, hunters) collected tiger feces samples on their routes. All tiger footprints found during the survey were tracked for 150–200 meters on the path for the presence of tiger feces. The staff members worked on routes inside Reserves and National Parks. This survey was organized by “Amur tiger center” that later transferred all collected samples to A.N. Severtsov Institute of Ecology and Evolution. No special permission is required in Russia for the tiger feces collection and to work with the endangered/protected species if they are realized non-invasively. In Russia, samples were collected in air temperatures below 0 C less than 7 days after defecation. Anthropogenic disturbance in Amur tiger range varied but was found to be higher in the south of the range [35]. There is no precise information on the number of tourists in each spot, but in Ussuriisky reserve (situated in southern area) it was about 2 persons/day. Although it may be higher in non-protected areas it would not be more than 30–40 people/day.

Bengal tiger samples were collected in three tiger reserves (Bandhavgarh, Kanha, Sariska) during January to March 2013–2015. In India, only fresh tiger samples (less than 1 day old) were used for this analysis. The permission to collect fecal samples from tiger was obtained from The Principal Chief Conservator of Forests, Government of Madhya Pradesh (Ref. no.2351 dated 14/04/2013) and the Chief Wildlife Warden, Rajasthan Forest Department, Rajasthan and the National Tiger Conservation Authority, Ministry of Environment and Forests, Government of India (Reference letters No. 2(3)/2005-PT dated 4.4.2008). In case of Bandhavargh and Kanha disturbance data were collected on the number of vehicles entering the park per day from the forest department. About 244,179 people have visited BTR (395 people/day) and KTR (509 people/day) during tourist season (Tyagi et al., in press). The disturbance level in Sariska Tiger Reserve was even higher and 32 villages (with about 10000 citizens) were situated inside the Tiger Reserve [23]. About 20% core area is being used for tourism in all tiger reserves of India. Samples were collected from core tiger tourism areas. All tigers in Sariska were equipped with the collars and sampling was conducted on the route of the individual (often–near the tiger kill of the known individual) [23]. In two other tiger reserves, the analysis of feces (at species level) has been conducted using molecular-genetic methods. Tiger samples were identified using three mitochondrial markers namely NADH5 subunit [36], Tig490 and Tig509 [37]. Previously, the genomic DNA was extracted using a Qiagen Stool Kit following the manufacturer’s protocol. The extracted DNA was stored in elution buffer and DNA was quantified using a Nanodrop-Spectrometer. Only tiger feces samples were analyzed for FGCM concentration.

Feces samples aliquots (5–50 g) were labelled (date, GPS-coordinates, species, sex and age (if possible)) and frozen (-18°C) until extraction (Table 1; S1 Table). All samples collected in Russia were extracted using three different methods with future comparison in mind. The first method has been used in Russian labs for many years and is based on extraction of wet samples by 90% methanol (WM, Wet samples, Methanol) [10, 21]. We added 0.9 ml of 90% methanol to 0.1 g of feces, shook it 30 minutes using an Ekros shaker (Ekros-Analitika, Russia), then centrifuged it for ten minutes at 1000 g. The supernatant was diluted with distilled water (1:1, v:v) [10]. It was done because concentrated methanol (90%) may have a negative effect on ELISA reactions. Samples were stored at -18°C until measurements. Since two other methods (see below) operate with FGCM concentration calculated per 1 g of dry feces, we also recalculate the WM results per 1 g of dry feces. For this we weighed an aliquot of each feces sample, dried it overnight at T = 80–90°C, and weighed again to determine the humidity. Final concentration was recalculated on 1 g of dry feces. The second method was used before in Indian labs based on ethanol extraction of dry feces (DE, Dried samples, Ethanol) [23, 24]. All feces samples were dried at T = 50°C (16 hours) in a dry dark place (using Techne Sample Concentrator (Techne, Staffordshire, UK)), powdered, 5 ml of 90% ethanol was added to 0,2 g feces and boiled for 20 minutes. Then samples were centrifuged at 500 g (10 minutes), the supernatant was transferred to another clear tube. The pellet was resuspended in 5 ml of 90% ethanol, vortexed for 1 minute, recentrifuged and pooled the supernatants. The supernatant was dried up at T = 40°C, then re-suspended in 1 ml of 100% methanol and sonicated for 30 s (Branson Ultrasonics 250, CT, USA). These methanol extracts were diluted with the distilled water (1:1.5 v/v to get 40% methanol solution). Fecal extracts were stored at -18°C until the assayed. The third extraction method (DM, Dried samples, Methanol) was identical to the first one (WM), however all samples were dried before extraction at T = 50°C (16 hours) with Techne Sample Concentrator. Then we added 0.9 ml of 90% methanol to 0.1 powdered dry feces and repeated the protocol of WM.

**Table 1:** Detailed protocols of feces collection and extraction for tiger samples. In the first row we show the country where samples were collected and the method of extraction.

Step	Russia/WM	Russia/DM	Russia/DE	India/DE
1	Samples collection and labelling	Samples collection and labelling	Samples collection and labelling	Samples collection and labelling
2	Freezing at -18°C	Freezing at -18°C	Freezing at -18°C	Freezing at -18°C
3	Transfer of frozen samples to Russian lab	Transfer of frozen samples to Russian lab	Transfer of frozen samples to Russian lab	Transfer of frozen samples to Indian lab
4		Thawing and drying feces at +50°C	Thawing and drying feces at +50°C	Thawing and drying feces at +50°C
5		Powdering feces	Powdering feces	Powdering feces
6	Thawing and weighing 0,1 g of wet feces	Weighing 0,1 g of dry feces	Weighing 0,2 g of dry feces	Weighing 0,2 g of dry feces
7	Addition of 0,9 ml of 90% Methanol	Addition of 0,9 ml of 90% Methanol	Addition of 5 ml of 90% Ethanol	Addition of 5 ml of 90% Ethanol
8	Shaking for 30 min	Shaking for 30 min	Boiling for 20 min	Boiling for 20 min
9	Centrifuged for 10 min	Centrifuged for 10 min	Centrifuged for 10 min	Centrifuged for 10 min
10			Taking all supernatant to clear tube	Taking all supernatant to clear tube
11			Resuspending pellets in 5 ml of 90% ethanol, vortex for 1 min	Resuspending pellets in 5 ml of 90% ethanol, vortex for 1 min
12			Centrifuged for 10 min	Centrifuged for 10 min
13			Combine both supernatants	Combine both supernatants
14			Evaporate supernatants (+40°C)	Evaporate supernatants (+40°C)
15	Taking 0,2 ml of supernatant to clear tube	Taking 0,2 ml of supernatant to clear tube	Add 100% methanol	Add 100% methanol
16	Dilute it with distilled water v/v 1:1	Dilute it with distilled water v/v 1:1	Dilute with distilled water v/v 1:1.5	Dilute with distilled water v/v 1:1.5
17	Keep frozen (-18°C) till measurements	Keep frozen (-18°C) till measurements	Keep frozen (-18°C) till measurements	Keep frozen (-18°C) till measurements
18	Weighing other aliquote of feces sample (0,5–3 g)			
19	Drying of feces at +90°C overnight			
20	Weighing the dry feces to calculate sample humidity			
	Recalculation of GC concentration on 1 g of dry feces	Recalculation of GC concentration on 1 g of dry feces	Recalculation of GC concentration on 1 g of dry feces	Recalculation of GC concentration on 1 g of dry feces

Measurements were conducted by EIA in the lab in Moscow on plate reader Multiscan EX (Thermo Labsystems, China) using commercial kits for cortisol (“ImmunoFA-Cortisol”, Cat № IF-03-01, Immunotek, Moscow, Russia). Cross-reactivity of antibodies was 6% to prednisolone and less than 1% for all other tested steroids. These kits were previously validated to measure cortisol metabolites in tigers [21].

All samples collected in Indian tiger reserves were assayed in Indian lab at the Centre of Cellular and Molecular Biology (Hyderabad) using the above kits on ELISA plate reader (Thermo Multiskan Spectrum Plate Reader, version 2.4.2, Thermo Scientific, Finland). All Indian samples were extracted through the DE method [23]. The extraction efficiency may vary among the labs but we strictly followed the protocol [23] and extraction efficiency was between 85–90% in both labs. We believe that it is acceptable to compare the results.

Statistical comparison of FGCM concentration at different study sites was conducted using the Mann-Whitney test, for the same samples extracted through different methods–using the Wilcoxon matched-pairs test. Moreover, a correlation analysis was carried out to compare different extraction methods.

## Results

**Fig 1:**
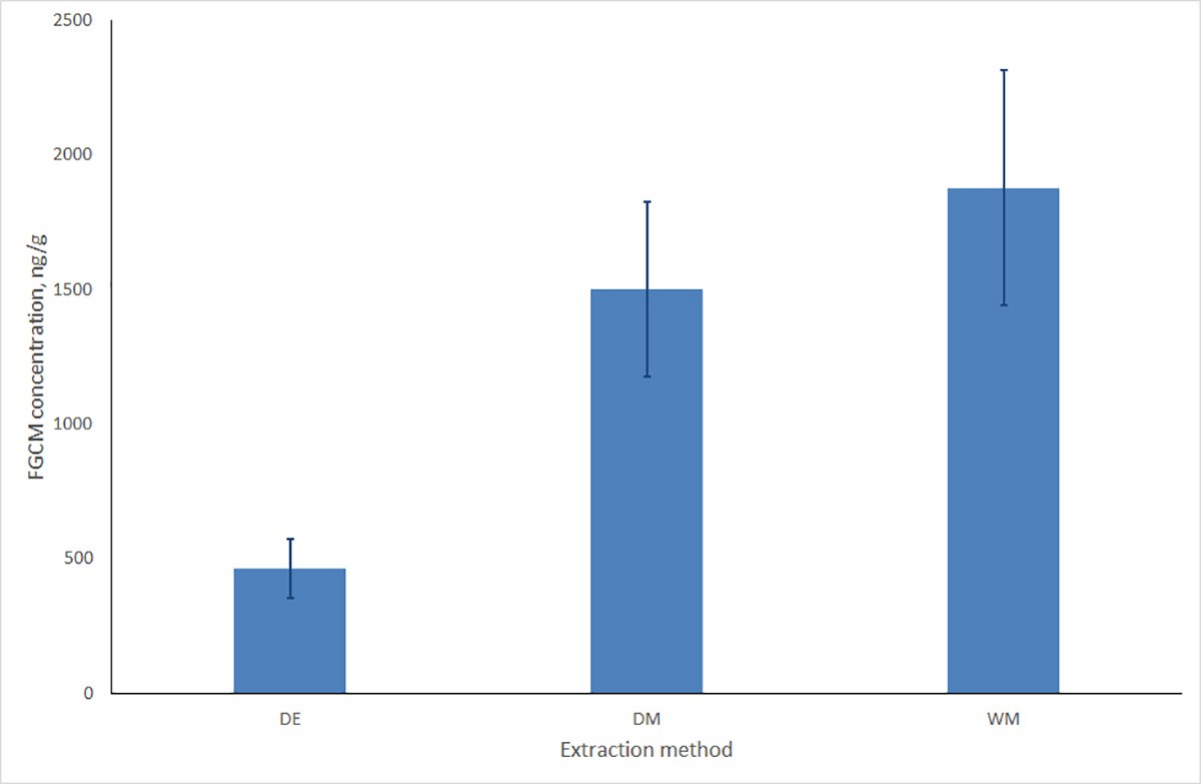
FGCM concentration in feces samples extracted by three different methods (DE–dried ethanol, DM–dried methanol, WM–wet methanol).

We analyzed 35 samples of Amur tiger extracted using three different methods. The average humidity of samples was 63±3%. FGCM concentration differed significantly between all three methods (Fig 1). The highest FGCM concentration was obtained using the WM method (1878±435 ng/g), an extraction by DM method gave significantly lower results (1500±324 ng/g; Wilcoxon test Z = 5.16; p<10^−6^), boiling with ethanol (DE) resulted in the lowest concentrations (501±125 ng/g; W-test Z = 5.13; p<10^−6^).

**Fig 2:**
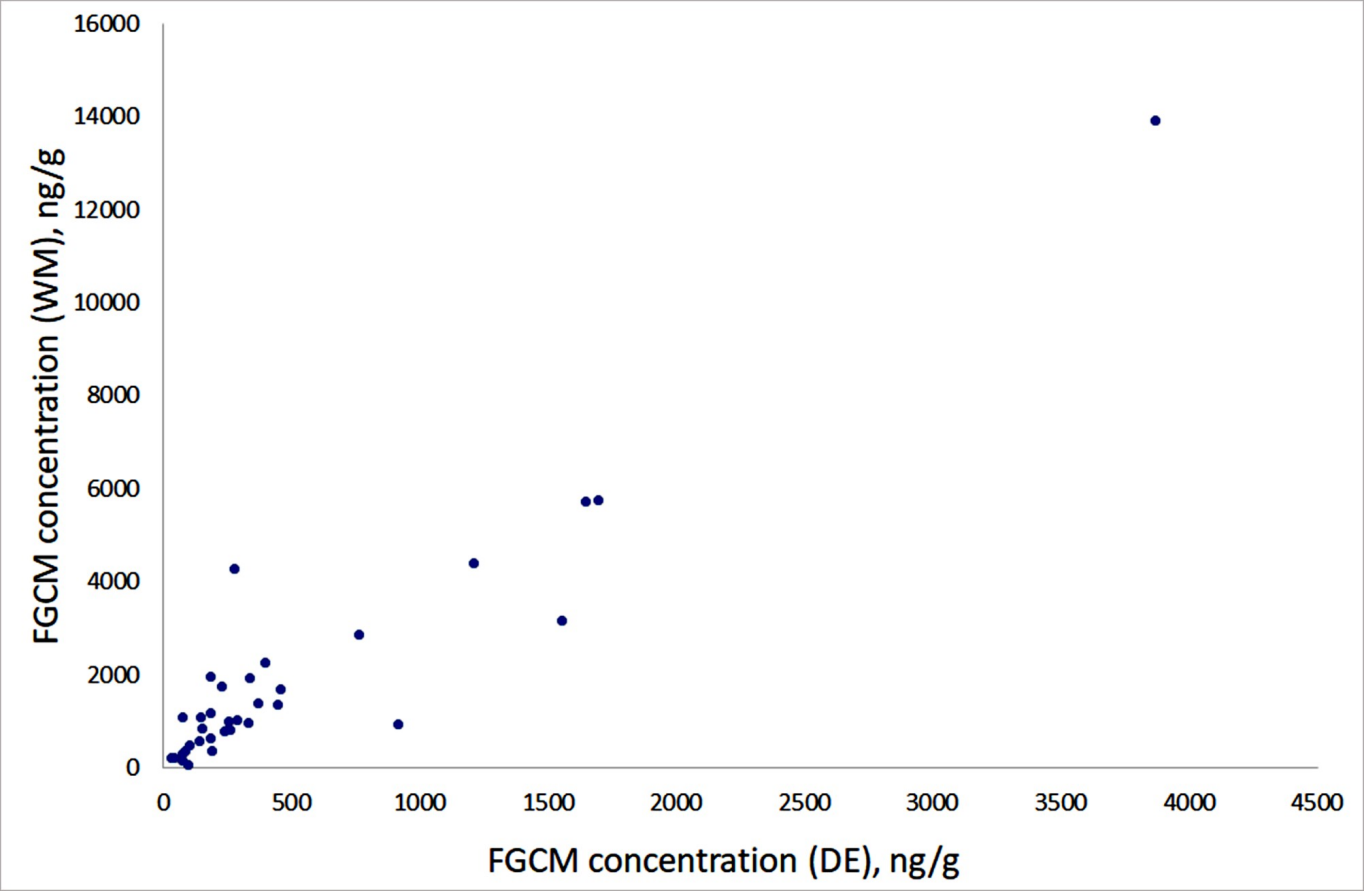
FGCM concentration in feces samples extracted by DE (dried ethanol) and WM (wet methanol) method (correlation r = 0.94).

However, FGCM concentrations in the same samples extracted through different means correlated significantly with each other (r = 0,89–0,94 for each pair of methods). The highest correlation coefficient was for WM and DE methods (Fig 2), the lowest for DE and DM methods.

**Fig 3:**
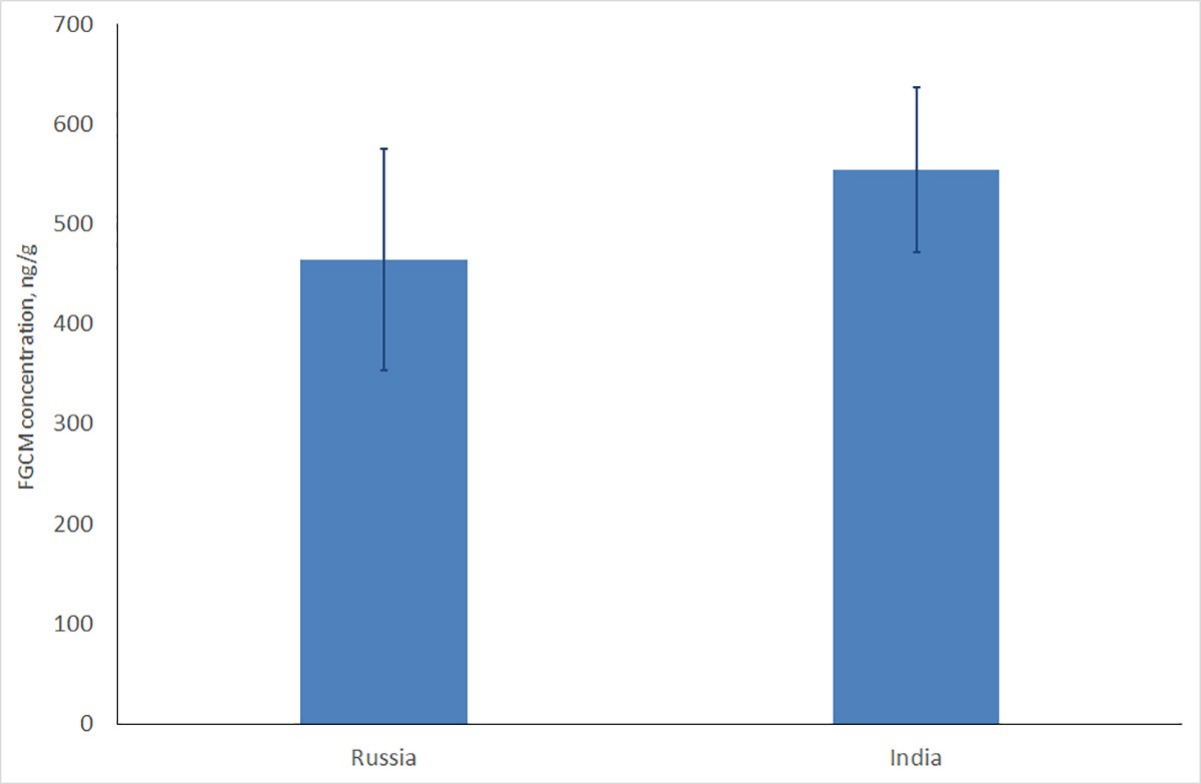
FGCM concentration in tigers’ feces in Russia and India.

For the samples collected in India only DE method of extraction has been used. Our sample size did not show any significant differences of FGCM level in three Indian Tiger Reserves. The FGCM level was the highest in Kanha Tiger Reserve (716±184 ng/g; n = 40) but it did not differ significantly from tigers’ FGCM levels in Bandhavargh and Sariska tiger reserves (respectively 383±37 ng/g, n = 40, Mann-Whitney test: Z = 1,00; ns, and 578±127 ng/g, n = 15, Z = 0,53; ns). Respectively, all these data were combined in one data set to compare it with the tigers FGCM level in Russia. Comparison of the FGCM in Bengal and Amur tigers was conducted only for samples that were extracted using the DE method. FGCM concentration was significantly higher in Bengal tigers than in Amur tigers (about 20%) (M-W test: Z = 2,55; n_1_ = 95; n_2_ = 40; p = 0,01) (Fig 3). We have to note that Sariska and Kahna tigers had significantly higher level of FGCM than tigers in Russia (respectively, M-W test: Z = 2,04; n_1_ = 15; n_2_ = 40; p = 0,04 and M-W test: Z = 2,20; n_1_ = n_2_ = 40; p = 0,03), while Bandhavargh tigers had level of FGCM similar with the tigers in Russia (Z = 1,85; n_1_ = n_2_ = 40; ns).

## Discussion

The application of different antibodies for non-invasive monitoring of hormonal status makes difficult to compare results from different labs. Application of different antibodies such as corticosterone [24], cortisol [21] and 11-oxoethiocholanolone [17] to estimate glucorticoids in the fecal sample have yielded different concentrations thus the values are unfit comparison. Moreover, even when antibodies were produced to the same antigen their cross-reactivity would result in different values. In Eurasian lynx, feces testosterone concentrations differed in two folds when different antibodies were used, although these values correlated significantly [10]. Thus, comparison of hormone data which come from different antibodies and difference extraction methods would yield no quantifiable or worthwhile results. However, a few studies have compared, for example, FGCM level of tigers in Australian zoos [38] was compared with Russian wild tigers [21] and some felids from US zoos [21, 39].

The other source of variation in values are the differences in extraction methods. Usually the extraction of steroids of mammalian species is based on alcohol (ethanol, methanol) of different concentrations (80–90%) and different procedures (shaking, centrifugation, boiling, evaporation, buffer dilution). Although the efficiency of extraction is mainly estimated by addition of native steroids (testosterone, cortisol, etc), it is necessary to note that for felids we usually work with metabolites of hormones and extraction efficiency for them may be very different to that of native hormones. It was clearly demonstrated in this study that application of different extraction methods results in 3-4-folds differences in concentrations. The highest concentrations were obtained when we extracted wet samples with 90% methanol (WM). Earlier it was shown that various methanol solutions provide higher hormones concentration than 100% ethanol [40]. It was also speculated [41] that extraction of dry feces may be more precise because in case of wet samples uncontrolled water may change alcohol concentration and its extraction ability. However, in our case with WM method with the samples humidity 40 or 80% and dilution 1:9 (feces:90% methanol) alcohol concentration did not change significantly (86% and 88% respectively) and did not affect hardly the extraction ability. Partly it is supported by the high correlation between the FGCM concentrations extracted from wet and dry samples (WM, DM). Higher WM concentration allowed us to hypothesize that drying of samples at +50C (especially by boiling) results in the destruction of some FGCM metabolites in feces. High correlations of values obtained using different methods demonstrated that all of them can be used for the FGCM measurements. It allowed us to compare the concentrations of FGCM in Bengal and Amur tigers. The highest concentration of FGCM was obtained using the WM method, methods with dry samples showed lower results. One of the explanations is that 12–16 hours (overnight or more) of drying may result in destruction of some steroids/their metabolites. However, this method is used in many labs of the world [15, 19, 23], although it may lead to inexact results.

Welfare level of animals, as seen through their FGCM level, may be affected by different factors. First of all, there are biotic factors such as food availability, predators presence, anthropogenic disturbances, etc [13, 41, 42]. The effect of abiotic factors was rarely tested in wildlife [21]. For Amur tiger low air temperature correlates with the increase of FGCM level [35]. This level was much higher in winter than in other periods [21]. Based on these data we hypothesis that that in winter FGCM level in Amur tiger will be much higher than in Bengal ones. Firstly, we compared FGCM level in Bengal tigers at all study sites in India. We did not discover any significant differences depending on the place, and we pooled all data on Bengal tigers together. Previous study showed the high stress level of tigers in Sariska tiger reserve [23], but possibly small sample size did not allow us to confirm or deny it in this research. We did not focus on the differences in FGCM level between these study sites (as well as the factors affecting it), because it is a goal of other study (Tyagi et al., in press). Most importantly, all data for Bengal tigers were pooled in one dataset. The actual situation turned out to be significantly different from our hypothesis: the Bengal tigers had higher FGCM level than Amur tigers in winter.

We hypothesis that high anthropogenic stress might be the reason for higher FGCM level in Bengal tigers. India is one of the most populated countries over the world (human population density is 402.8 individuals/km^2^). In Russian Far East (inhabited by the Amur Tiger) human density is extremely low (10 individuals /km^2^). In India tigers’ home ranges are significantly smaller than in Russia [32, 33], they are situated in tigers’ reserves and national parks which are visited regularly by tourists. Number of visitors/tourists was very different between Russian (less than 40, in reserves 2–5 persons/day) and Indian (395–509) study sites. Anthropogenic stress (short distance from villages) may increase stress level in Carnivores [43, 44], including Bengal tigers and even lead to some problems with their reproductive abilities [22]. Possibly, visitors effect on Tiger reserves and National Parks may increase stress level and decrease welfare of tigers in India. The effect of visitors on FGCM level was described for other carnivores (grey wolf, *Canis lupus* [45]).

The second hypothesis associates high cortisol level in Bengal tigers with the higher tiger population density in this region in comparison to Russian Far East. The density of tigers is many times higher in India (for example, a density of 11.33 individuals /100 km^2^ was described for Wayanad Wildlife sanctuary, Kerala [46] while in Russia, in Ussuriisky reserve, tigers’ density approximates 0.15 individuals /100 km^2^) [47]. Normally tigers of the same sex may compete for the territory and high density of conspecifics may result in higher cortisol level. However, this hypothesis, while supported by the data on solitary living rodents [48, 49], was never tested for carnivores.

To sum up, Bengal tigers had higher FGCM level than Amur tigers in winter (dry season in India). Many factors may affect stress/welfare level of animals. Increase of FGCM level may be caused by anthropogenic pressure, sexual intercourse, hunger, low air temperatures, conflicts with conspecifics or sympatric species. Bengal tigers had much higher density of potential prey than Amur ones, with supposed hunger (limitation of food) being less probable in India. Winter temperatures are much lower in Russian Far East (-10-40°C) than in India, so this factor does not seem to affect FGCM level in Bengal tigers. Both subspecies have food competitors among sympatric species of carnivores, however, in India all of them are much smaller than tigers although in Russia brown bears (*Ursus arctos*) may be dangerous to the Amur tigers at the Rissian Far East. Most likely, the higher level of glucocorticoids in Bengal tigers might be due to high anthropogenic disturbance [23] or/and high density of tigers, which will result in higher contacts with the conspecifics (including aggressive and sexual contacts) or their marking points or footprints. However, a detailed investigation is required to understand the reasons of this phenomenon.

## Supporting information

S1 Table Detailed protocols of feces collection and extraction for tiger samples. (DOCX)

S1 File The concentrations of glucocorticoids metabolites in tigers’ feces samples. (XLSX)
