# Supplementary material for: Comparison of tigers' fecal glucocorticoids level in two extreme habitats
Source: PLoS One. 2019 Apr 10;14(4):e0214447. doi: 10.1371/journal.pone.0214447 (PMC6457496; doi:10.1371/journal.pone.0214447)
Supplement: S1 Table — (DOCX) [file pone.0214447.s001.docx]

Table 1. Detailed protocols of feces collection and extraction for tiger samples. In the first row, we show the country where samples were collected and the method of extraction.

| Step | Russia/WM | Russia/DM | Russia/DE | India/DE |
| --- | --- | --- | --- | --- |
| 1 | Samples collection and labelling | Samples collection and labelling | Samples collection and labelling | Samples collection and labelling |
| 2 | Freezing at  -18°C | Freezing at -18°C | Freezing at -18°C | Freezing at -18°C |
| 3 | Transfer of frozen samples to Russian lab | Transfer of frozen samples to Russian lab | Transfer of frozen samples to Russian lab | Transfer of frozen samples to Indian lab |
| 4 | Thawing and weighing 0,1 g of wet feces | Thawing and drying feces at +50°C | Thawing and drying feces at +50°C | Thawing and drying feces at +50°C |
| 5 | Addition of 0,9 ml of 90% Methanol | Powdering feces | Powdering feces | Powdering feces |
| 6 | Shaking for 30 min | Weighing 0,1 g of dry feces | Weighing 0,2 g of dry feces | Weighing 0,2 g of dry feces |
| 7 | Centrifuged for 10 min | Addition of 0,9 ml of 90% Methanol | Addition of 5 ml of 90% Ethanol | Addition of 5 ml of 90% Ethanol |
| 8 | Taking 0,2 ml of supernatant to clear tube | Shaking for 30 min | Boiling for 20 min | Boiling for 20 min |
| 9 | Dilute it with distilled water v/v 1:1 | Centrifuged for 10 min | Centrifuged for 10 min | Centrifuged for 10 min |
| 10 | Keep frozen  (-18°C) till measurements | Taking 0,2 ml of supernatant to clear tube | Taking all supernatant to clear tube | Taking all supernatant to clear tube |
| 11 | Weighing other aliquote of feces sample (0,5-3 g) | Dilute it with distilled water v/v 1:1 | Resuspending pellets in 5 ml of 90% ethanol, vortex for 1 min | Resuspending pellets in 5 ml of 90% ethanol, vortex for 1 min |
| 12 | Drying of feces at +90°C overnight | Keep frozen  (-18°C) till measurements | Centrifuged for 10 min | Centrifuged for 10 min |
| 13 | Weighing the dry feces to calculate sample humidity | Recalculation of GC concentration on 1 g of dry feces | Combine both supernatants | Combine both supernatants |
| 14 | Recalculation of GC concentration on 1 g of dry feces |  | Evaporate supernatants (+40°C) | Evaporate supernatants (+40°C) |
| 15 |  |  | Add 100% methanol | Add 100% methanol |
| 16 |  |  | Dilute with distilled water v/v 1:1.5 | Dilute with distilled water v/v 1:1.5 |
| 17 |  |  | Keep frozen  (-18°C) till measurements | Keep frozen  (-18°C) till measurements |
| 18 |  |  | Recalculation of GC concentration on 1 g of dry feces | Recalculation of GC concentration on 1 g of dry feces |
